# Interactions between the Gut Microbiome and Mucosal Immunoglobulins A, M, and G in the Developing Infant Gut

**DOI:** 10.1128/mSystems.00612-19

**Published:** 2019-11-26

**Authors:** Anders Janzon, Julia K. Goodrich, Omry Koren, the TEDDY Study Group, Jillian L. Waters, Ruth E. Ley

**Affiliations:** a Department of Microbiome Science, Max Planck Institute for Developmental Biology, Tübingen, Germany; b Azrieli Faculty of Medicine, Bar Ilan University, Safed, Israel; University of California San Diego

**Keywords:** gut microbiome, infant, diabetes, immunoglobulins, IgA, IgM, IgG, antibody coating, infant gut development, FACS, host response, immunology, microbial ecology

## Abstract

Interactions between the gut microbiome and immunoglobulin A (IgA) in the gut during infancy are important for future health. IgM and IgG are also present in the gut; however, their interactions with the microbiome in the developing infant remain to be characterized. Using stool samples sampled 15 times in infancy from 32 healthy subjects at 4 locations in 3 countries, we characterized patterns of microbiome development in relation to fecal levels of IgA, IgG, and IgM. For 8 infants from a single location, we used fluorescence-activated cell sorting of microbial cells from stool by Ig-coating status over 18 months. We used 16S rRNA gene profiling on full and sorted microbiomes to assess patterns of antibody coating in relation to age and other factors. All antibodies decreased in concentration with age but were augmented by breastmilk feeding regardless of infant age. Levels of IgA correlated with relative abundances of operational taxonomic units (OTUs) belonging to the *Bifidobacteria* and *Enterobacteriaceae*, which dominated the early microbiome, and IgG levels correlated with *Haemophilus*. The diversity of Ig-coated microbiota was influenced by breastfeeding and age. IgA and IgM coated the same microbiota, which reflected the overall diversity of the microbiome, while IgG targeted a different subset. *Blautia* generally evaded antibody coating, while members of the *Bifidobacteria* and *Enterobacteriaceae* were high in IgA/M. IgA/M displayed similar dynamics, generally coating the microbiome proportionally, and were influenced by breastfeeding status. IgG only coated a small fraction of the commensal microbiota and differed from the proportion targeted by IgA and IgM.

**IMPORTANCE** Antibodies are secreted into the gut and attach to roughly half of the trillions of bacterial cells present. When babies are born, the breastmilk supplies these antibodies until the baby’s own immune system takes over this task after a few weeks. The vast majority of these antibodies are IgA, but two other types, IgG and IgM, are also present in the gut. Here, we ask if these three different antibody types target different types of bacteria in the infant gut as the infant develops from birth to 18 months old and how patterns of antibody coating of bacteria change with age. In this study of healthy infant samples over time, we found that IgA and IgM coat the same bacteria, which are generally representative of the diversity present, with a few exceptions that were more or less antibody coated than expected. IgG coated a separate suite of bacteria. These results provide a better understanding of how these antibodies interact with the developing infant gut microbiome.

## Abstract (precis)

Antibodies are secreted into the gut and attach to roughly half of the trillions of bacterial cells present. When babies are born, the breastmilk supplies these antibodies until the baby’s own immune system takes over this task after a few weeks. The vast majority of these antibodies are IgA, but two other types, IgG and IgM, are also present in the gut. Here, we ask if these three different antibody types target different types of bacteria in the infant gut as the infant develops from birth to 18 months old and how patterns of antibody coating of bacteria change with age. In this study of healthy infant samples over time, we found that IgA and IgM coat the same bacteria, which are generally representative of the diversity present, with a few exceptions that were more or less antibody coated than expected. IgG coated a separate suite of bacteria. These results provide a better understanding of how these antibodies interact with the developing infant gut microbiome.

## INTRODUCTION

The gut microbiota, the immune system, and their interactions develop in tandem in infancy (1, 2). The immunoglobulin A (IgA) component of breast milk is protective against infection in infants and may also direct the development of the gut microbiota. IgA is secreted into the gut lumen, where it binds antigens from food and microbiota, thereby excluding them from direct contact with the host epithelial cells (3). At birth, neonates generally have undetectable IgA in meconium (4), and it takes a few weeks for their immune systems to initiate IgA production and secretion into the gut (5). Breastmilk is an important early source of IgA, and breastfeeding is associated with high levels of fecal IgA in infants (4, 6). Planer et al. characterized the fraction of IgA-coated fecal microbiota in infants over the first few years of life and reported differences between breastmilk- and formula-fed infants, which may relate to differences in how the microbiota develop in these two groups (7).

Although IgA is the dominant antibody in the gut, IgM and IgG are also present. The juvenile gut sees up to 5 g of secretory IgA daily, 100-fold less secretory IgM, and 1,000-fold less IgG (8). IgM and IgA are both produced by B cells locally, and the predominant class switching that occurs in B cells of the gut-associated lymphoid tissue is from IgM to IgA. Both are secreted into the gut via the same mechanism (polymeric Ig receptor), IgM as a pentamer and IgA as a dimer. In contrast, IgG is the most common antibody in circulation but can also be transported into the gut via a neonatal Fc receptor (9). Whereas IgA/M are produced in response to luminal microbial epitopes that are sampled by dendritic cells, IgG induction is thought to require crossing of the barrier by antigens, such that IgG is not produced continuously in response to common gut antigens.

Based on its similarity to IgA, IgM may be expected to follow similar patterns of microbiota binding, whereas IgG may not. In healthy adults, IgA has been shown to coat a greater proportion of the stool microbiota than IgG or IgM, but whether the diversity of taxa targeted by these antibodies differs has not been reported (10). To gain a baseline understanding of how IgA, IgG, and IgM coat gut microbiota during microbiome development in infancy, here we performed a longitudinal analysis of the fecal microbiome of healthy infants in which we characterized the diversity of microbiota coated with IgA, IgM, and IgG as a function of time and with respect to feeding regimen and antibody levels.

## RESULTS

### The small sample set is representative of the larger TEDDY population.

**FIG 1:**
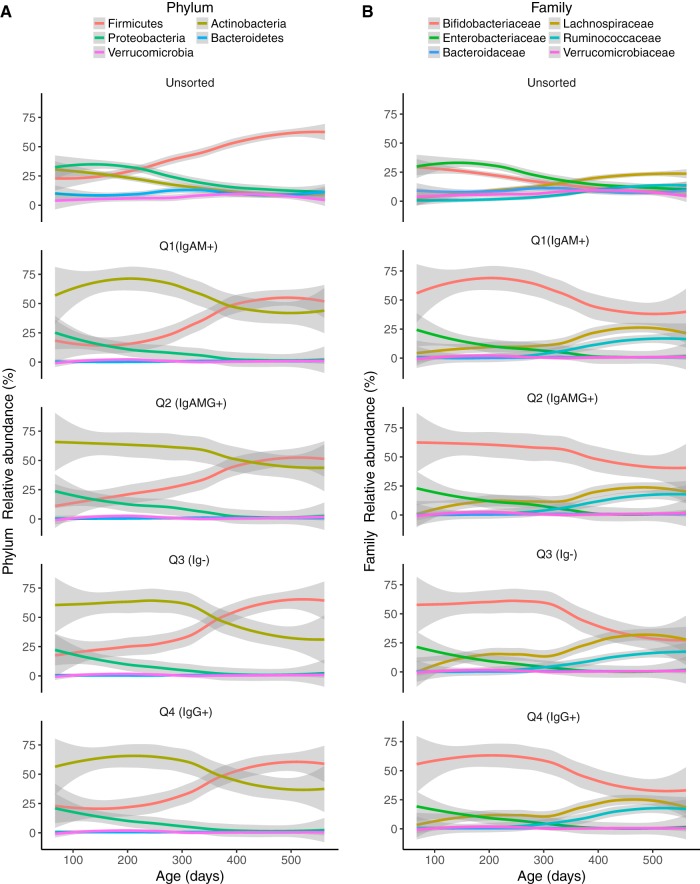
Changes in microbial composition over time. (A and B) Percent relative abundance of the dominant bacterial phyla (A) and families (B) in the unsorted samples (top plot in each panel). Each of the four quadrants is plotted over time. Q1 (IgAM+), IgA and IgM both high, IgG low; Q2 (IgAMG+), all high; Q3 (Ig−), all low; Q4 (IgG+), IgG high, IgA and IgM both low.

**FIG 2:**
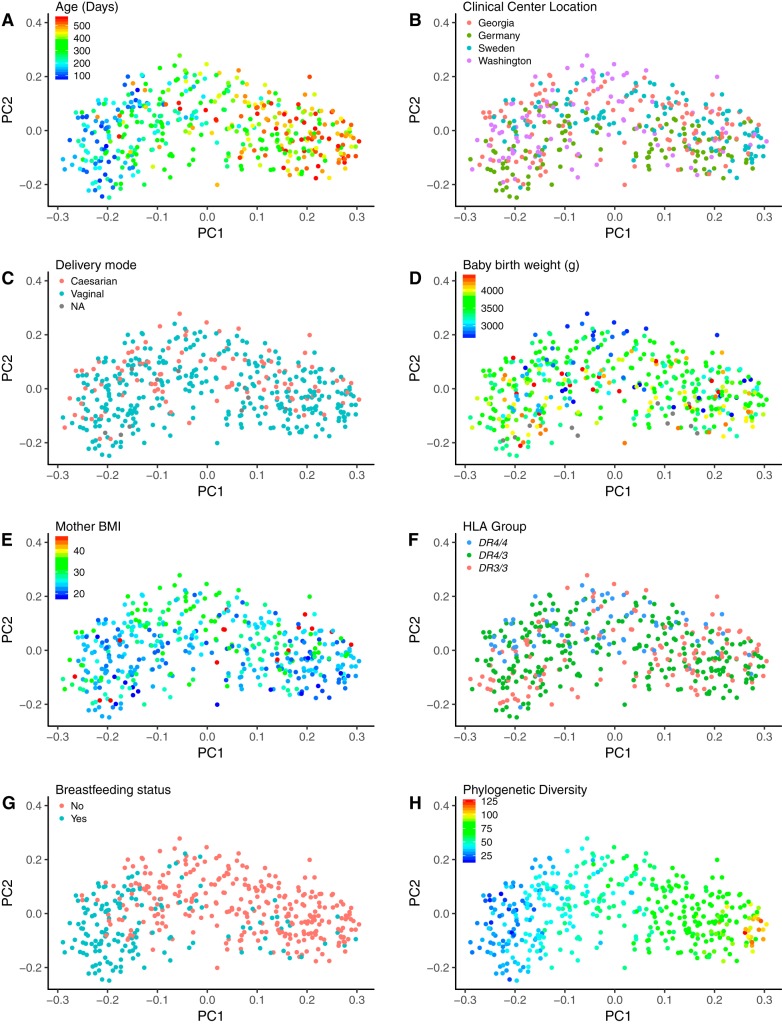
Patterns of beta diversity (using unweighted UniFrac) in 32 infant time series with covariates visualized. Shown are the first two axes (PC1 and PC2) from principal coordinate analysis of the unweighted UniFrac distances among the unsorted fecal microbiome samples of 32 infants over the first couple years of life. Points are colored by participant characteristics and phylogenetic diversity. (A) Infant age in days at the time of sampling. (B) Geographic location of the TEDDY-associated primary care center where samples were collected; this includes samples from Germany, Sweden, and the United States (Georgia and Washington). (C) Vaginal or cesarean delivery. (D) Baby birth weight in grams. (E) Body mass index (BMI) of the mother before pregnancy. (F) HLA genotypes of the infant were DR4-DQA1*030X-DQB1*0302/DR3-DQA1*0501-DQB1*0201 (*DR3/4*), DR4-DQA1*030X-DQB1*0302/DR4-DQA1*030X-DQB1*0302 (*DR4/4*), and DR3-DQA1*0501-DQB1*0201/DR3-DQA1*0501-DQB1*0201 (*DR3/3*). (G) Breastfeeding status at the time of sampling. (H) Faith’s phylogenetic diversity. In all plots colored by a quantitative trait, blue indicates lower values and red indicates higher values.

**FIG 3:**
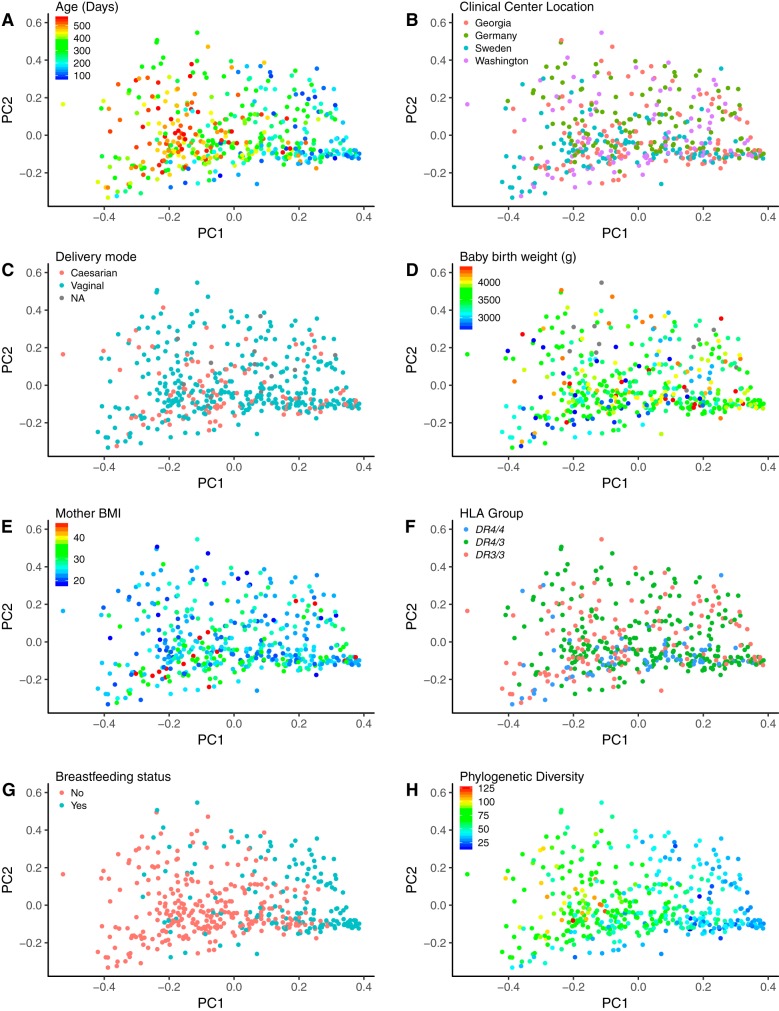
Patterns of beta diversity (using weighted UniFrac) in 32 infant time series with covariates visualized. Shown are the first two axes (PC1 and PC2) from principal coordinate analysis of the weighted UniFrac distances between the unsorted fecal microbiome samples of 32 infants over the first couple years of life. Points are colored by participant characteristics and phylogenetic diversity. (A) Infant age in days at the time of sampling. (B) Geographic location of the TEDDY-associated primary care center where samples were collected; this includes samples from Germany, Sweden, and the United States (Georgia and Washington). (C) Vaginal or cesarean delivery. (D) Baby birth weight in grams. (E) Body mass index (BMI) of the mother before pregnancy. (F) HLA genotypes of the infant were DR4-DQA1*030X-DQB1*0302/DR3-DQA1*0501-DQB1*0201 (*DR3/4*), DR4-DQA1*030X-DQB1*0302/DR4-DQA1*030X-DQB1*0302 (*DR4/4*), and DR3-DQA1*0501-DQB1*0201/DR3-DQA1*0501-DQB1*0201 (*DR3/3*). (G) Breastfeeding status at the time of sampling. (H) Faith’s phylogenetic diversity. In all plots colored by a quantitative trait, blue indicates lower values and red indicates higher values.

**FIG 4:**
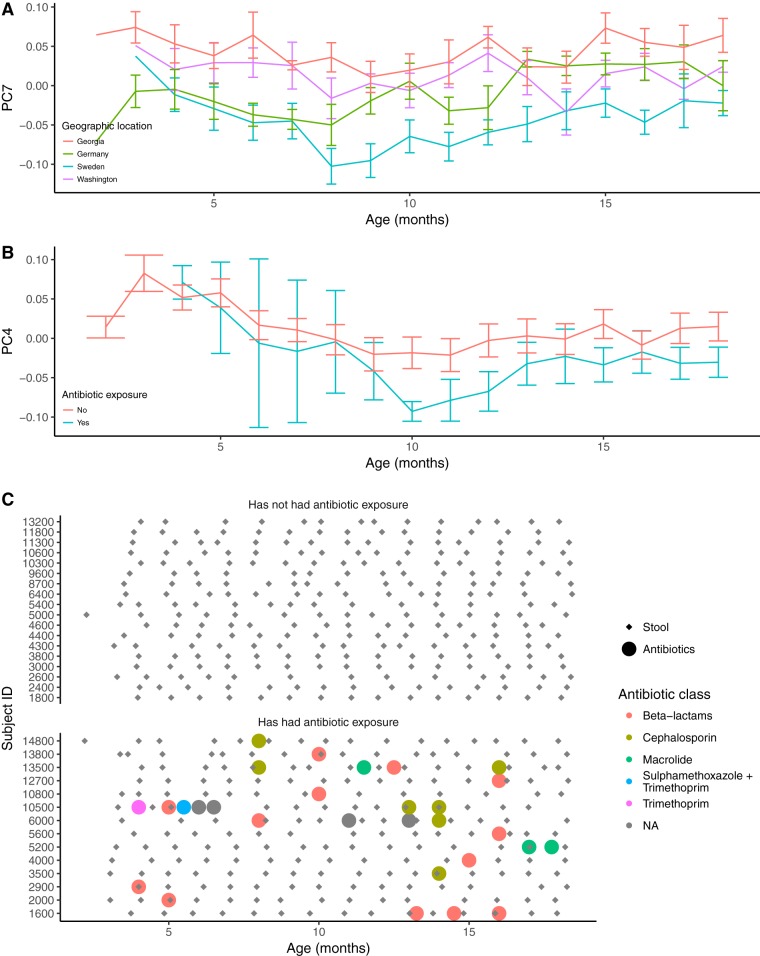
Beta diversity measures in relation to age. (A and B) PC4 (A) or PC7 (B) of unweighted UniFrac distances across time points. Lines show the mean PC values ± SEM grouped by time point and geographic location (A) or previous antibiotic exposure (B). (C) Plot illustrating the time of stool collection and antibiotic use for each participant. NA, not applicable.

We looked for previously reported patterns of microbiota diversity in relation to covariates in order to establish that the small cohort used here (32 subjects sampled longitudinally, unsorted stool) is representative of the larger TEDDY cohort. More information on the study participants is shown in Table 1. Two recent papers have reported on the gut microbiome in the TEDDY cohort: Stewart et al. (11) employed a 16S rRNA gene survey on 903 subjects, and Vatanen et al. (12) used metagenomics with 783 subjects. Our results recapitulate those of Stewart et al. and Vatanen et al. in the following ways: (i) *Bifidobacteriaceae* and *Enterobacteriaceae* dominated the infant gut at early time points, while *Firmicutes* increased in relative abundance later (Fig. 1); (ii) breastfeeding status was significantly associated with between-sample diversity (e.g., beta diversity, as observed from associations between breastfeeding status and several principal coordinates [PCs] from both unweighted and weighted UniFrac principal coordinate analysis [PCoA]; Fig. 2 and 3; see also Table S1 in the supplemental material) after correcting for multiple testing; (iii) the only other factor with a significant association with beta diversity was geographic location (Fig. 4A); (iv) we observed a weak association between antibiotic exposure and beta diversity (unweighted UniFrac PC4; Fig. 4B and C); (v) age had a significant association with microbiome richness (Chao1, Faith’s phylogenetic diversity, observed species, and Gini coefficient; all *P* < 10^−10^). We observed that age and breastfeeding status were associated with microbiome diversity, corroborating the findings of previous studies (11, 12), and therefore the subset of subjects that we used in subsequent analysis are representative of the larger TEDDY cohort.

**TABLE 1:** Characteristics of study participants^*a*^

Parameter	Value for study participant			
	No. sampled	Means ± SEM	Median	Range
Baby birth weight (g)	31	3,526.34 ± 80.33	3,505	2,620–4,480
Mother body mass index	32	26.26 ± 1.08	24.34	17.83–47.3
Gestational age (wk)	32	39.81 ± 0.23	40	37–42.14
Mother weight gain (kg)	32	15.24 ± 1.06	14.45	5.91–28.6
Maternal age	32	33.31 ± 0.99	31.5	22–43
Paternal age	31	34.74 ± 0.79	34	27–43

a Location, Georgia (USA, *n* = 8), Washington (USA, *n* = 8), Germany (*n* = 8), and Sweden (*n* = 8); delivery mode, Caesarian (*n* = 8), vaginal (*n* = 23), unknown (*n* = 1); sex, female (*n* = 16) and male (*n* = 16).

10.1128/mSystems.00612-19.2 TABLE S1 Associations of covariates with alpha and beta diversity metrics of the unsorted 16S data. Download Table S1, XLSX file, 0.01 MB. Copyright © 2019 Janzon et al. 2019 Janzon et al. This content is distributed under the terms of the Creative Commons Attribution 4.0 International license.

### Stool antibody levels decrease with infant age and are related to breastfeeding status.

We observed that levels of IgA, IgG, and IgM (measured by enzyme-linked immunosorbent assay [ELISA] in the same stool samples for which the microbiome was analyzed) were positively correlated to each other across the sample set (Fig. S1). Age (and therefore also microbiome alpha diversity) was negatively correlated with levels of IgA (repeated measures correlation [*r_rm_*] = −0.45, *P* = 6.14 × 10^−22^), IgG (*r_rm_* = −0.37, *P* = 1.13 × 10^−14^), and IgM (*r_rm_* = −0.23, *P* = 3.29 × 10^−5^), although the IgG and IgM correlation with age was not as strong as that for IgA (Fig. S1). No association of geographic location or HLA genotype was observed with any of the immunoglobulin levels in stool.

10.1128/mSystems.00612-19.6 FIG S1 Levels of IgA, IgG, and IgM are all positively correlated with each other and negatively correlated with age and alpha diversity. The figure shows box plots of the within-individual Pearson correlation coefficients between each of the following variables: age, unweighted UniFrac (PC1, PC2, and PC3), phylogenetic diversity, IgA (log transformed), IgG (log transformed), and IgM (log transformed). The box plots are colored by the repeated measures correlation (as calculated by rmcorr R package) and illustrate the agreement between the mean within-individual Pearson correlation coefficient and the repeated measures correlation for each pair of variables. Download FIG S1, PDF file, 0.2 MB. Copyright © 2019 Janzon et al. 2019 Janzon et al. This content is distributed under the terms of the Creative Commons Attribution 4.0 International license.

**FIG 5:**
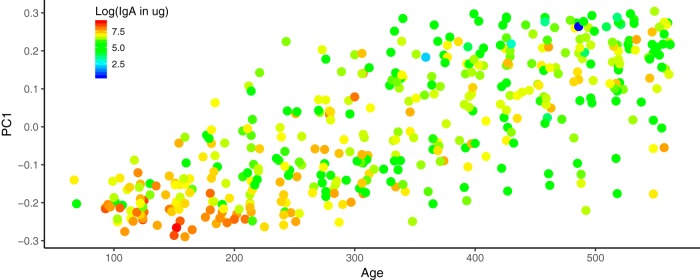
Infant fecal IgA concentrations over the first couple years of life. PC1 from the PCoA of the fecal microbiome unweighted UniFrac distances is plotted against the age of the infant in days at the time of sampling. The points are colored by the log-transformed fecal IgA concentrations; lower concentrations are blue and higher concentrations are red.

**FIG 6:**
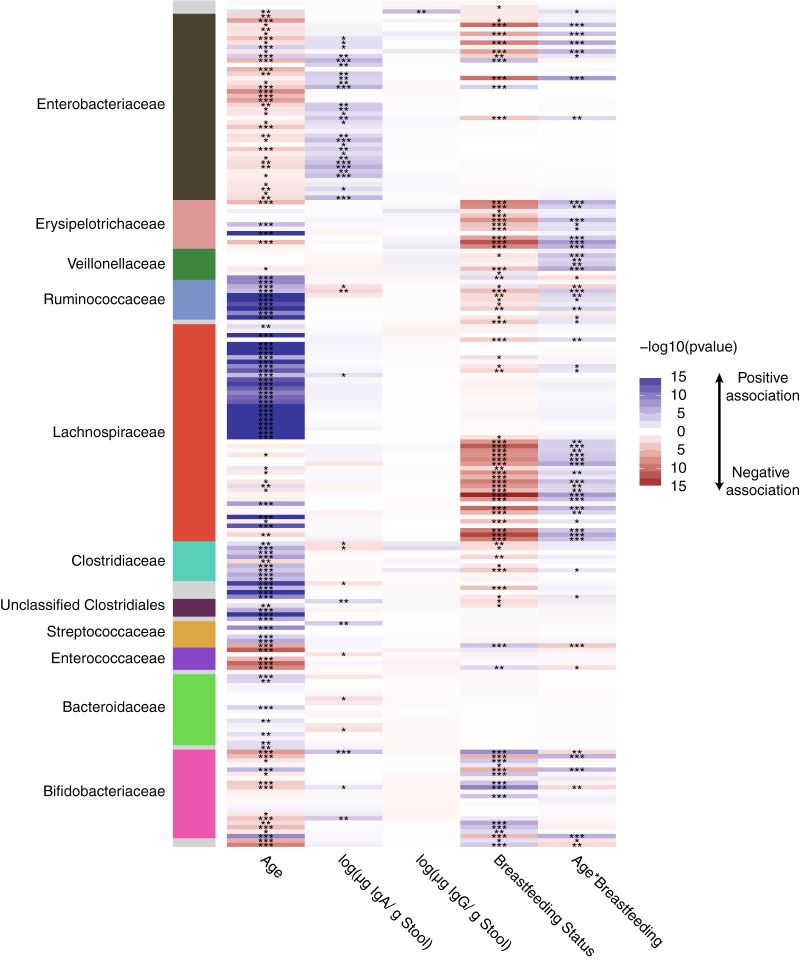
Associations of age, fecal IgA and IgG concentrations, and breastfeeding status with common OTUs in the infant fecal microbiomes. Heatmap showing, along the vertical axis, each of the common OTUs (nonzero value in >40% of samples tested) and, along the horizontal axis, each of the fixed factors examined. The heatmap is colored by the –log10 of the *P* value from a linear mixed model examining the OTU association with the fixed effects. Blue indicates a positive association and red a negative association, while white represents a *P* value of 1; the darker the color, the more significant the association. The panel on the left is colored by the family-level taxonomic association of the OTU.

We assessed the relationships between stool IgA levels, age, and breastfeeding status to ask how IgA levels varied over time. We were particularly curious to see if the cessation of breastfeeding correlated with stool IgA levels and if the effect of breastfeeding was age dependent. We observed that IgA levels in stool decreased significantly with age (*P* = 1.79 × 10^−11^) (Fig. 5 and 6). Furthermore, we observed that breastfeeding was significantly associated with higher stool IgA levels (*P* = 0.039), and that this association was not dependent on the age of the infants. These observations corroborate previous reports that IgA levels decrease with age, but that at any age, breastmilk delivers additional IgA to the infant.

We next examined the association between stool levels of IgA, IgG, and common OTUs (e.g., those shared by greater than 40% of samples) (Fig. 6). IgA levels were positively associated with several *Enterobacteriaceae* OTUs and a few *Bifidobacteriaceae* OTUs. Only one OTU was associated with IgG levels in feces. This OTU belonged to genus *Haemophilus* (Benjamini-Hochberg [BH] adjusted *P* = 1.66 × 10^−3^).

### IgA and IgM coat many of the same microbial cells.

**FIG 7:**
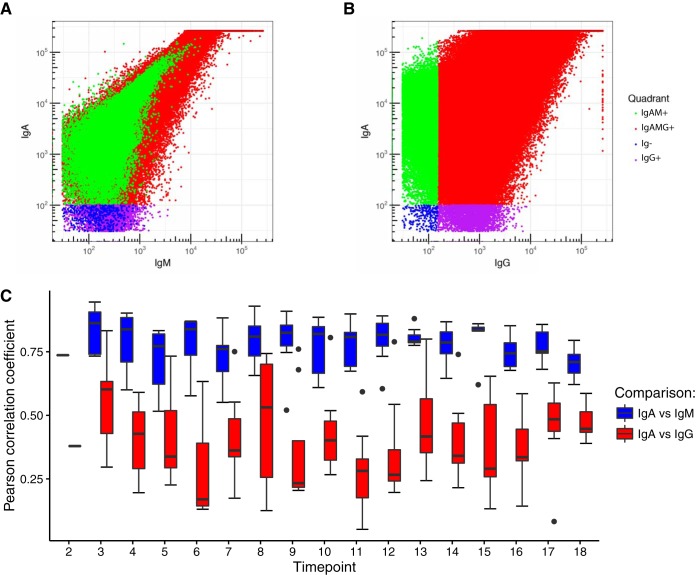
IgA and IgM coat many of the same microbial cells. (A) Representative data for FACS sorting of microbial cells in infant fecal samples. Frequencies of IgM- versus IgA-tagged cells (green and red, respectively). (B) This panel shows how the four quadrants were gated. IgAM+, IgA and IgM both high; IgAMG+, all three high; Ig−, all three low; IgG+, IgG high, others low. (C) Box plots at each time point of the Pearson correlations (*y* axis) between IgA and IgM signals and between IgA and IgG signals from the FACS sorting of each fecal sample.

For 8 infants from Georgia, for whom 15 time points sampled over 18 months were available, we sorted cells according to their antibody-coating status using FACS based on their distributions in four quadrants (Qs) (Text S1). The gatings for these four populations and the resulting quadrants are illustrated in Fig. 7A and B. We used heavy-chain-specific antibodies that do not cross-react with other classes of Ig (Text S1); thus, the patterns observed by flow cytometry indicate that many microbial cells were tagged with multiple antibodies. The gating patterns indicate that IgA and IgM coat many of the same cells, since the patterns of coating overlap. IgG-coated cells, on the other hand, fell into three categories: IgAM+, those low in IgG and high in IgA/IgM; IgAMG+, those highly coated in IgG, IgA, and IgM; Ig−, uncoated cells; and IgG+, those highly coated in IgG and low in IgA/IgM. We observed that the correlation between IgA and IgM coating was consistently higher than the correlation between IgA and IgG coating across sampling time points (*P* < 10^−10^) (Fig. 7C).

10.1128/mSystems.00612-19.1 TEXT S1 Extended materials and methods and list of members of the TEDDY study group. Download Text S1, DOCX file, 0.03 MB. Copyright © 2019 Janzon et al. 2019 Janzon et al. This content is distributed under the terms of the Creative Commons Attribution 4.0 International license.

### Specific taxa discriminate total and FACS-sorted populations.

**FIG 8:**
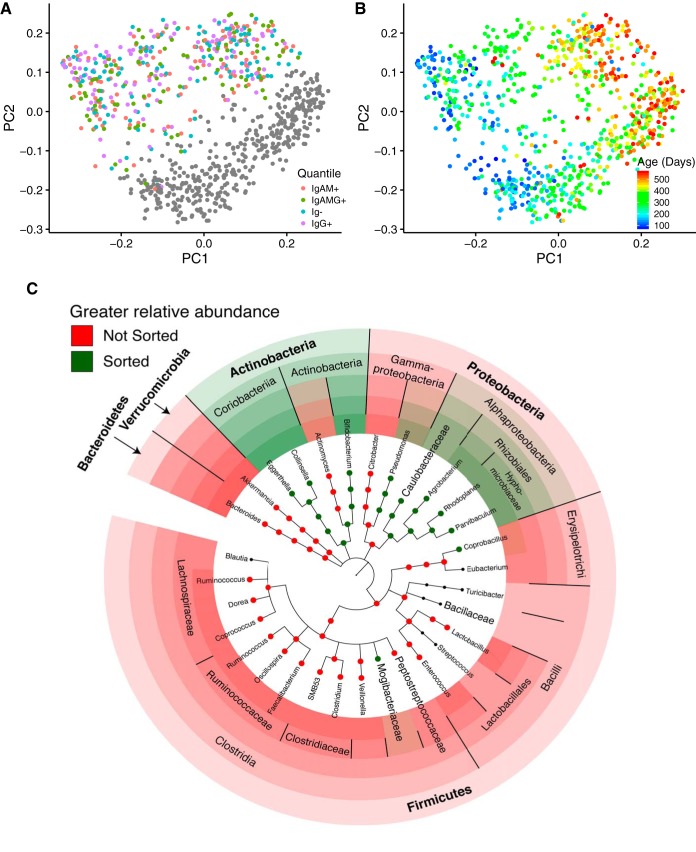
Fecal microbiome composition is altered by FACS but remains significantly associated with infant age. (A) PCoA of unweighted UniFrac distances colored by FACS quadrant. Unsorted samples are shown in gray. (B) The same PCoA plot of unweighted UniFrac distances colored by participant age. (C) Summary of taxa that are differentially abundant between the total (red) and FACS (green) populations.

We compared the diversity of the sorted microbiota (all Qs combined) to that of the whole microbiota (unsorted) to assess the impact of the FACS process on microbial diversity. We observed that the microbiota composition of the sorted cells (all Qs) is distinct from that of the total microbiome: a combined PCoA analysis of unweighted UniFrac analysis shows clear separation of unsorted and sorted cells along PC2 (Fig. 8A). The unsorted population was richer (Chao1, phylogenetic diversity, and observed species) and exhibited greater evenness (Gini coefficient) than all sorted cells (*P* < 10^−10^ for all metrics) (Fig. S2). We applied linear mixed models to identify taxa that were differentially abundant between the unsorted and sorted populations (all Qs). This analysis identified members of the *Bacteroidetes*, *Verrucomicrobia*, *Gammaproteobacteria*, and most *Firmicutes* as comparatively enriched in the unsorted fraction and *Actinobacteria* and *Alphaproteobacteria* as enriched in the sorted fraction (Fig. 8C). The difference in composition between sorted and unsorted fractions likely stems from the FACS process itself; for example, cells belonging to *Actinobacteria* and *Alphaproteobacteria* may be less likely to clump than others. Thus, the difference between sorted and unsorted microbiomes introduces the caveat that subsequent analyses with the sorted data are performed on a subset of the microbiome.

10.1128/mSystems.00612-19.7 FIG S2 Alpha diversity differs between the sorted and unsorted fraction of the gut microbiome. Each panel shows how the alpha diversity metric (Chao1, panels A and E; phylogenetic diversity, panels B and F; observed species, panels C and G; Gini coefficient, panels D and H) changes over time. The lines indicate the means ± SEM within a time point and either geographic location (A to D) or FACS quadrant (E to H). Download FIG S2, PDF file, 0.2 MB. Copyright © 2019 Janzon et al. 2019 Janzon et al. This content is distributed under the terms of the Creative Commons Attribution 4.0 International license.

### Antibody-targeted cells exhibit patterns similar to those of the total population.

As observed for unsorted cells, the majority of the variation in the sorted cells (PC1 of the unweighted UniFrac PCoA) was explained by age (Fig. 8B). Similarly, as age increased, alpha diversity also increased (Fig. S3). Analysis of variance indicated that FACS quadrant was significantly associated with most of the first 10 PCs from the PCoA (restricted to only sorted 16S rRNA gene data) of both unweighted and weighted UniFrac and all four alpha diversity metrics. *Post hoc* pairwise comparisons showed that this association was driven primarily by a difference between IgG+ (high IgG only) and the other three quadrants. This finding might represent an overall diversity difference between the high IgG-only cells and others. However, the number of cells sorted into the high-IgG (IgG+) quadrant was significantly lower than those of the other three quadrants (Fig. S3). Indeed, most of the cells coated in IgG were also coated by IgA and IgM and are therefore sorted into IgAMG+ rather than IgG+. This significant difference in cell number between the quadrants could explain the difference in diversity observed between IgG+ and the other three quadrants.

10.1128/mSystems.00612-19.8 FIG S3 Percentage of the sorted microbiome fraction by FACS quadrant. For each subject we quantified the percentage of the sorted fecal microbiome that was detected in each of the 4 quadrants. Sample IDs are listed along the *y* axis, with relative abundance shown along the *x* axis and colored by FACS quadrant. Download FIG S3, PDF file, 0.2 MB. Copyright © 2019 Janzon et al. 2019 Janzon et al. This content is distributed under the terms of the Creative Commons Attribution 4.0 International license.

After exclusion of IgG+ (high-IgG only), we observed some significant associations between the first 10 PCs of the beta diversity PCoAs and the FACS quadrant, as well as several associations with infant age and infant breastfeeding status. The significant associations with unweighted UniFrac were with age (PCs 1, 2, and 3), breastfeeding status (PCs 1, 2, 3, 6, and 7), and FACS quadrant (PCs 7 and 10) (Table S2). Further analysis of the FACS quadrant associations showed that Ig− (uncoated) was different from both IgAM+ (*P* = 0.030) and IgAMG+ (*P* = 0.001) along PC7, and that IgAMG+ differed from Ig− (*P* = 0.010) along PC10 (*post hoc* pairwise comparisons between the quadrants using Tukey’s honestly significant difference [HSD] method to adjust for multiple comparisons). Among the weighted UniFrac PCs, many are associated with FACS quadrant (PCs 1, 5, 7, 8, and 9; *post hoc* analysis shows that Ig− is different from IGAM+ and IgAMG+) and PC1 is associated with breastfeeding status and PC2 with age (Table S2). These results indicate that the diversity of cells targeted by antibodies is influenced by breastfeeding status and infant age. Furthermore, the specific combination of antibodies on the cells is not random.

10.1128/mSystems.00612-19.3 TABLE S2 Associations of covariates with alpha and beta diversity metrics with age, FACS quadrant, and breastfeeding status in FACS 16S data. Download Table S2, XLSX file, 0.01 MB. Copyright © 2019 Janzon et al. 2019 Janzon et al. This content is distributed under the terms of the Creative Commons Attribution 4.0 International license.

### Specific taxa vary in their antibody-coating profiles.

**FIG 9:**
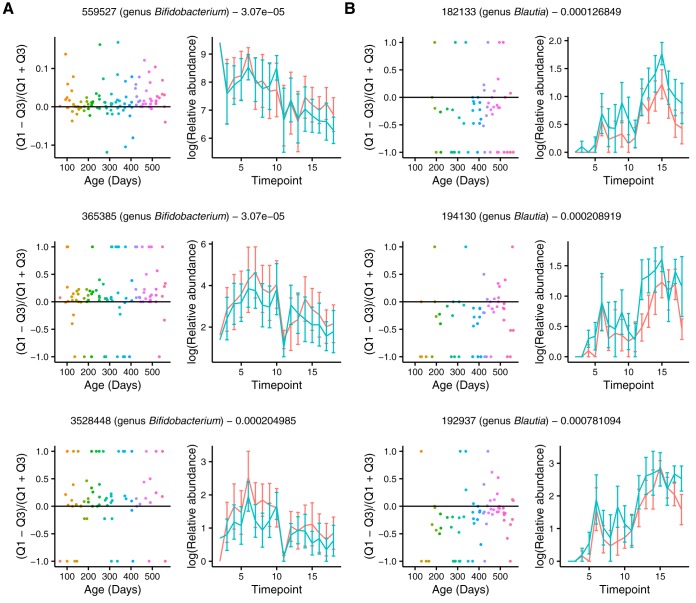
Many differentially abundant OTUs between quadrants 1 (IgAM+, IgM and IgA both high) and quadrant 3 (Ig−, low coating) are classified as *Blautia* and *Bifidobacterium.* (A and B) Plots for a subset of differentially abundant OTUs between Q1 and Q3 illustrating higher relative abundance of *Bifidobacterium* OTUs in Q1 (IgAM+) (A) and higher relative abundance of *Blautia* OTUs in Q3 (B). Plots on the left show the log(Q1 OTU abundance) − log(Q3 OTU abundance) divided by log(Q1 OTU abundance) + log(Q3 OTU abundance) over time, where each point represents a sample and is colored by time point. Positive values on the *y* axis indicate enrichment of the OTU abundance in Q1 (IgAM+), and negative values indicate enrichment of the OTU abundance in Q3 (Ig−); this is similar to the IgA index defined in Planer et al. (7). Plots on the right show the average of the log-transformed OTU relative abundance in Q1 (IgAM+, red) and Q3 (Ig−, blue) at each time point. OTU Greengenes ID, taxonomic classification, and *P* value from the linear mixed model using OTU as a response variable are indicated above each set of graphs. Only common OTUs (nonzero value in >40% of samples tested) were tested.

We next identified specific OTUs differentially abundant between the quadrants. To identify common OTUs (i.e., OTUs with nonzero sequence counts in greater than 40% of FACS samples examined) with differential relative abundance between quadrants, we performed linear mixed models using each OTU as a response variable. We searched for differences between the coated (IgAM+ and IgAMG+; excluding IgG+ because of low cell population) and uncoated (Ig−) populations. When comparing IgAM+ to Ig− Qs, we observed significant differences in the relative abundances of 80 out of 190 OTUs (Fig. 9 and Table S3). These mostly included OTUs classified as *Blautia*, which had higher relative abundance in Ig− (uncoated) than other Qs. OTUs that were higher in IgAM+ (IgM and IgA both high) were mostly classified as *Bifidobacterium*, unclassified *Enterobacteriaceae*, and Ruminococcus gnavus. Similarly, 101 OTUs had differential relative abundances between IgAMG+ (all high) and Ig− (all low), with *Blautia* being significantly higher in Ig− (Table S4).

10.1128/mSystems.00612-19.4 TABLE S3 Comparison of OTU relative abundances between FACS quadrant 1 (IgM and IgA both high) and FACS quadrant 3 (all low coating). Download Table S3, XLSX file, 0.1 MB. Copyright © 2019 Janzon et al. 2019 Janzon et al. This content is distributed under the terms of the Creative Commons Attribution 4.0 International license.

10.1128/mSystems.00612-19.5 TABLE S4 Comparison of OTU relative abundances between FACS quadrant 2 (all high coating) and FACS quadrant 3 (all low coating). Download Table S4, XLSX file, 0.04 MB. Copyright © 2019 Janzon et al. 2019 Janzon et al. This content is distributed under the terms of the Creative Commons Attribution 4.0 International license.

### Cross-validation for IgA-targeted microbiota.

We compared the results of our analysis with those of Planer et al., who used FACS followed by 16S rRNA gene sequencing to characterize the IgA coated microbes of 40 twin pairs over the first couple years of life (7). We used the same reference database as Planer et al. to classify sequences; therefore, we could compare OTUs directly. A total of 14 individual OTUs were identified by Planer et al. as being consistently targeted or not targeted by IgA (*n* = 5 and *n* = 9, respectively). Of these OTUs, two met the same criteria as those used in this study, in that they had a nonzero value in >40% of the samples. Interestingly, both OTUs behaved similarly with respect to antibody coating. Greengenes OTU 365385 (genus *Bifidobacterium*), consistently targeted by IgA in the Planer et al. study, was proportionally higher in both IgAM+ (BH adjusted *P* = 3.07 × 10^−05^) (Fig. 9A) and IgAMG+ (BH adjusted *P* = 1.88 × 10^−05^) than Ig−. The other Greengenes OTU detected in both data sets (606927; family *Peptostreptococcaceae*) was consistently nontargeted by IgA in the Planer et al. study, and similarly, we observed this OTU to have a higher relative abundance in Ig− (uncoated) than IgAMG+ (BH adjusted *P* = 0.013) (Table S4). These comparisons indicate that taxon-specific antibody-coating profiles can be generalizable across studies.

## DISCUSSION

Interactions between IgA and the gut microbiome in the developing gut are important for health. Indeed, low levels in IgA coating of the gut microbiome in infants is associated with development of allergies and asthma during childhood (13) and with Crohn’s disease in children (14). Although the dynamics of IgA levels in the infant gut over time, and in response to breastfeeding, are well characterized, the IgA-microbiota coating, and those of the other antibodies in the gut (IgM and IgG), are less well understood. This study provides a baseline view of how levels of IgA, IgG, and IgM correlate with age and other factors over the first 18 months of life in healthy Western children and details how specific taxa are targeted by these antibodies over time in a subset of children.

This study included infants from four distinct geographic locations, two in the United States and two in Europe. Infants from the different locations had slightly different microbiota, as previously reported (11, 12). Effects of breastfeeding status on the microbiome and antibody levels, and the decrease in antibody levels with age, were also similar across subjects, regardless of the shift in diversity associated with the geographic location of the infants. We also observed a strong impact of breastfeeding status and age on gut microbial community structure, as previously reported (11, 12). In contrast, we did not observe an effect of birth mode on microbiome diversity in this data set.

Our longitudinal analysis of the Georgia infants’ gut microbiota targeted by antibodies over time revealed that IgM and IgA target the same microbial populations. IgA and IgM targeting of the same cell population implies these two antibodies can be induced via the same mechanism, and that, like IgA, IgM induction is a local phenomenon. In contrast, we observed patterns of IgG coating quite distinct from those of IgA and IgM. IgG is induced as a result of the systemic immune system’s recognition of a “non-self” antigen and is not, in general, produced locally in the gut. Thus, whereas IgA/M responses mirror the gut microbiome generally, the bacterial targets of IgG are related to those targeted by the systemic immune system around the time of collection. We noted a fair amount of overlap between the IgA/M- and IgG-coated fractions, however, suggesting redundancy across all three classes of antibody.

The majority of IgA secreted to the gut is polyclonal and thought to be relatively unselective, as it binds with epitopes that are widely shared among gut bacteria (15, 16). In addition, secretory IgA affinity maturation by somatic hypermutation is a prominent feature of the human repertoire (17), and a large fraction of secretory IgA may be targeted to specific bacterial species (18). Using a reversible colonization system in germfree mice, Hapfelmeier and colleagues showed that a specific strain of E. coli induced a sustained IgA response even after the strain was no longer there (>100 days), but that colonization with other bacteria induced a decline in the Escherichia coli-specific IgA and an increase in IgA response to the newly introduced bacteria (19). These results suggested the presence of a long-lived compartment of IgA-secreting plasma cells in the intestinal *lamina propria* but that the compartment has a finite size, so that the IgA secreted into the intestine depends on the dominant luminal microbial species. Based on this model, we would expect the majority of the IgA-coated fraction of microbiota to mirror the unsorted microbiota (although a small subset of taxa exhibit either more or less coating than expected based on this model; see below), and allowing for the caveats of FACS sorting, this is the general pattern that we observed.

A few OTUs provided exceptions to the general pattern, in that some OTUs were more highly represented in the IgA-coated than uncoated (Ig−) cell populations. In particular, OTUs classified as *Bifidobacterium*, unclassified *Enterobacteriaceae*, and Ruminococcus gnavus had higher representation in the IgA-coated cell fraction. In addition, we observed that levels of IgA antibody in stool were correlated to a few *Bifidobacteriaceae* OTUs and several *Enterobacteriaceae* OTUs. Planer et al. also observed one of the same OTUs was highly coated in IgA in the fecal samples of children from Malawi (7), and *Bifidobacteria* in particular have been shown to induce high levels of IgA in the gut (20). *Bifidobacteria* and *Enterobacteriaceae* dominated the infant gut microbiomes early on; however, the higher-than-expected levels of specific OTUs belonging to these taxa in the IgA-coated fraction suggests they are stimulating IgA production specific to them in excess of what is expected from their relative abundances in the microbiota. Alternatively, given that here we characterize antibody coating of bacteria in the feces, these taxa may be preferentially cleared from the intestine once coated with IgA. Antibody coating is implicated in aggregation, which may help retain bacteria in mucus, as well as clearance, because larger particles are more likely to be entrained; it is likely that both mechanisms operate simultaneously and differentially for various taxa.

In contrast to the high-IgA-coated microbiota, OTUs classified as *Blautia* showed a lack of antibody coating. In the healthy mouse cecum, specific OTUs have been shown to be less coated than what would be expected by chance (21). One explanation could be antibody evasion by regulation of epitopes in response to the antibodies. Certain bacterial species, including the gut commensal Bacteroides thetaiotaomicron, have been shown to downregulate the expression of epitopes in response to IgA (22–24). Alternatively, the relative undercoating of specific taxa in stool may reflect patterns of clearance, and here uncoated *Blautia* OTUs are more likely to be shed than their coated counterparts. How IgA coating relates to the microbial ecology of the microbiota, their growth rates, the immune response to specific epitopes, and ultimately shedding of the cells is complex and not well understood.

Although a large proportion of sorted cells was coated by all three antibodies, we did observe differences between the patterns of IgG coating and the patterns of IgA/IgM coating (note that the low cell count, approximately 5% of the total population, in the IgG-only coated category casts some doubt on the reliability of these results). One OTU was associated with IgG levels in feces; this OTU belonged to the genus *Haemophilus.* IgG is produced systemically in response to infection, and it is also produced in response to immunization. Thus, this pattern may have resulted from the *Haemophilus* vaccine, which is given to infants. It is one of the few vaccines against a bacterium, and it is not a mucosal vaccine. *Haemophilus* colonizes the upper respiratory mucosa, and not all are pathogenic. The *Haemophilus* that we observed here may include commensal species that share capsule epitopes with the vaccine strains.

One limitation of the present study is the relatively small sample sizes utilized, as only 32 infants were studied for the overall microbiome effects, whereas 8 infants were investigated for antibody-coating patterns over time. Although we recapitulate many results of the developing infant gut from the TEDDY cohort in the unsorted microbiome data with 32 samples, fewer studies have analyzed antibody coating of microbes within the infant microbiome. The 8 infants investigated with respect to antibody coating were selected from a single location; therefore, the patterns observed may not reflect what is seen in other populations. Nevertheless, our study provides a foundation toward further understanding how immune targeting of the gut microbiome may alter microbiome development.

Our results indicate similar dynamics of antibody levels and microbiome development with age and breastfeeding status in infants from different locations. Patterns of IgA/M patterning indicate that sorting for IgM-coated cells are not any more informative than sorting for IgA alone. IgG coating of the infant gut microbiome may reveal antigen exposure if gut microbes cross-react. These data provide a baseline reference for further investigation of healthy or unhealthy children’s gut microbiomes in infancy.

## MATERIALS AND METHODS

### Subjects and selection criteria.

This study was conducted using fecal samples collected by the international type-1 diabetes (T1D) epidemiological prospective cohort study, The Environmental Determinants of Diabetes in the Young (TEDDY) (25). Subjects were screened for T1D risk through HLA genotyping, and all samples used in this study were collected from children with HLA genotypes that put them at high risk of developing T1D. None of the subjects included in the current study have developed T1D or seroconverted for any of the 3 measured T1D-associated autoantibodies (glutamic acid decarboxylase autoantibodies, IA-2 autoantibodies, and insulin autoantibodies) by 24 months of age and had not developed T1D by December 2016 (approximately 10 years of age). We focused on a set of longitudinal stool samples (9 to 16 time points per subject) obtained from 32 age- and sex-matched healthy children (468 fecal samples) from the United States (Georgia and Washington), Germany, and Sweden. Subjects were excluded if they provided fewer than 12 longitudinal samples, if their participation and sample collection was through a long distance protocol, or if they dropped out of the study before or at 24 months of age. Additional data collected as part of the TEDDY study and that were used in the current analysis included physical descriptors (e.g., length and weight), diet, illnesses, hospitalizations, vaccinations and medicines, and social data, such as daycare attendance.

### Ethics approval and consent to participate.

The study was approved by U.S. institutional review boards and European ethics committee boards in the respective countries, which are the following: Georgia’s Medical College of Georgia Human Assurance Committee (2004 to 2010), Georgia Health Sciences University Human Assurance Committee (2011 to 2012), Georgia Regents University Institutional Review Board (2013 to 2015), Augusta University Institutional Review Board (2015 to present), Washington State Institutional Review Board (2004 to 2012) and Western Institutional Review Board (2013 to present), Germany’s Bayerischen Landesärztekammer (Bavarian Medical Association) Ethics Committee, Sweden’s Regional Ethics Board in Lund, Section 2 (2004 to 2012), and Lund University Committee for Continuing Ethical Review (2013 to present). Written informed consent was obtained for all study participants from a parent or primary caregiver.

### IgA, IgG, and IgM ELISAs.

IgA, IgG, and IgM were quantified in duplicate for all fecal samples from a subset of 8 infants from the Georgia study site. A serial dilution of reference human serum (Bethyl Laboratories, Montgomery, TX) was used for generating a standard curve, and blocking buffer was used as a negative control. The immunoglobulin concentrations were log transformed (with an offset of 0.01 added to IgM and IgG concentrations to handle zero values) before downstream analysis (see Text S1 in the supplemental material).

### FACS sorting of antibody-coated cells.

Microbial FACS was performed on 117 fecal samples derived from 8 subjects from the Georgia study site. Samples were vortexed and centrifuged to separate bacterial cells from large particles and debris, and the resulting supernatant was transferred to a new tube. This supernatant was then resuspended in phosphate-buffered saline (PBS) and centrifuged to remove unbound immunoglobulins. The resulting pellet was then resuspended in PBS plus 0.1% bovine serum albumin before labeling with the respective anti-IgA, anti-IgG, and anti-IgM fluorophore-labeled antibodies. Samples were incubated for 30 min and then washed twice prior to flow cytometry and cell sorting (see Text S1).

### Microbial diversity analysis.

Genomic DNA was extracted from 468 fecal samples (approximately 20 mg per sample) using the PowerSoil-htp DNA isolation kit (MoBio Laboratories Ltd., Carlsbad, CA). The 16S rRNA V4 hypervariable region was then PCR amplified (26), and the resulting amplicons were pooled and sequenced using the Illumina MiSeq 2× 250-bp paired-end platform at the Cornell Biotechnology Resource Center Genomics Facility.

Sequence analysis was performed using the open-source software package QIIME 1.8.0 (Quantitative Insights Into Microbial Ecology) (27). Briefly, reads were quality filtered before using open-reference OTU picking at 97% against the Greengenes August 2013 database. The data were rarefied at 18,429 sequences per sample, which was the lowest sample sequencing depth over 1,000, in order to include as many samples and sequences as possible (described in more detail in Text S1).

### Statistical analyses.

**(i) Linear mixed models.** All linear mixed-effects models were fit using the lme4 (28) package in R. Significance was assessed using an F-test with a Satterthwaite approximation for denominator degrees of freedom, calculated by the R package lmerTest. Post hoc pairwise comparisons were performed using Tukey’s HSD tests implemented in the lsmeans (29) R package.

**(ii) Association of subject characteristics with beta diversity and alpha diversity.** A linear mixed model was used for associating individual metadata with alpha and beta diversity. The only factors used in the model examining the effect of time since exposure to oral antibiotics on unweighted UniFrac PC4 were age, time since oral antibiotic exposure, and the random effect for subject.

**(iii) Correlations between immunoglobulin levels, age, and microbial diversity.** The R package rmcorr (30) was used to calculate pairwise repeated measures correlations (and their significance) between levels of IgA, IgG, and IgM, age, the first three unweighted UniFrac PCoA PCs, and phylogenetic diversity. A repeated measures correlation was used because it accounts for the multiple sampling of individuals.

For further details, see Text S1.

### Data availability.

Raw sequencing data are available from the European Nucleotide Archive using accession number PRJEB32930.

10.1128/mSystems.00612-19.9 FIG S4 Defining the gating strategy for FACS quadrants. Shown is the pregating strategy designed to eliminate sorting of cell multiplets and debris, before immunoglobulin-specific stains are gated and sorted into fractions. Panels to A to C show pregating steps to exclude events corresponding to particles with aberrant cell size or granularity by using dot plots of forward (FSC) and side scatter (SSC) area (-A), height (-H), and width (-W). Red polygons indicate the sort gates for the given population of cells within each panel. Parentheses by the population number indicate the percentage of cells that were used as input for the subsequent cell sorting step. (A) Population 1 (P1) side scatter area versus forward scatter area. (B) Population 2 (P2) forward scatter height versus width. (C) Population 3 (P3) side scatter height versus width. (D) Histogram of cytosolic cell stain DyeCycle Violet with a fluorescence threshold gate set to discriminate noncellular particles. Events in population P4, indicated by the red arrow, were then sorted into quadrants, as shown in panel E, and samples were collected from each quadrant. (E) Alexa Fluor 488 (IgA) versus APC (IgG). The percentages of P4 cells assigned to each quadrant are indicated in red text for each quadrant. Download FIG S4, PDF file, 1 MB. Copyright © 2019 Janzon et al. 2019 Janzon et al. This content is distributed under the terms of the Creative Commons Attribution 4.0 International license.

10.1128/mSystems.00612-19.10 FIG S5 Illustration of the relationship between the time point variable and the age of the participants. Sample collection from each participant started around 2 to 3 months of age, and subsequent samples were collected approximately each month following the initial sample. Some analyses relied on defining time points that relate to the age of the participant at the time of sampling and are also comparable across participants. The full method for assigning time points to a sample is described in Materials and Methods. (A and B) In both plots, each point indicates a stool sample, and the *x* axis shows the participant’s age in days at the time of sample collection. (A) The *y* axis is the time point variable used throughout the analyses. We see that there is minimal overlap in age between the time points. (B) The *y* axis is the participant’s anonymized ID, and this plot shows that across participants each time point reflects a similar age. Download FIG S5, PDF file, 0.2 MB. Copyright © 2019 Janzon et al. 2019 Janzon et al. This content is distributed under the terms of the Creative Commons Attribution 4.0 International license.
