# Supplementary figures and images for: Interactions between the Gut Microbiome and Mucosal Immunoglobulins A, M, and G in the Developing Infant Gut
Source: mSystems. 2019 Nov 26;4(6):e00612-19. doi: 10.1128/mSystems.00612-19 (PMC6880043; doi:10.1128/mSystems.00612-19)

Pearson correlation coefficient per individual

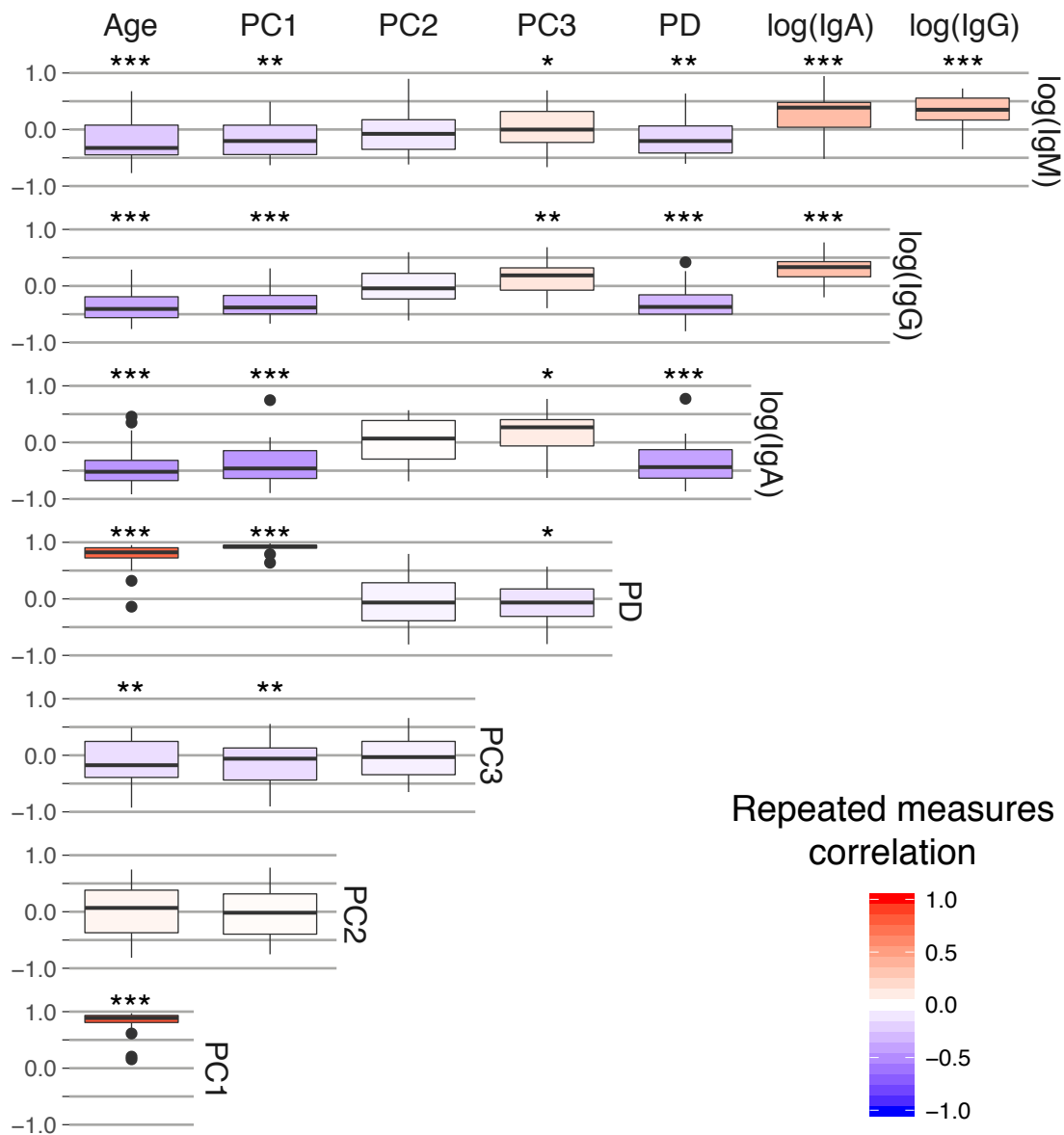

Supplement: FIG S1 [file mSystems.00612-19-sf001.pdf]

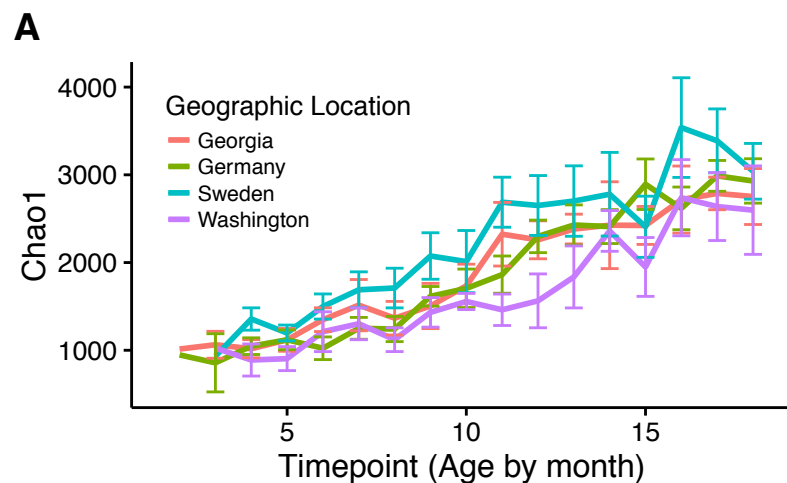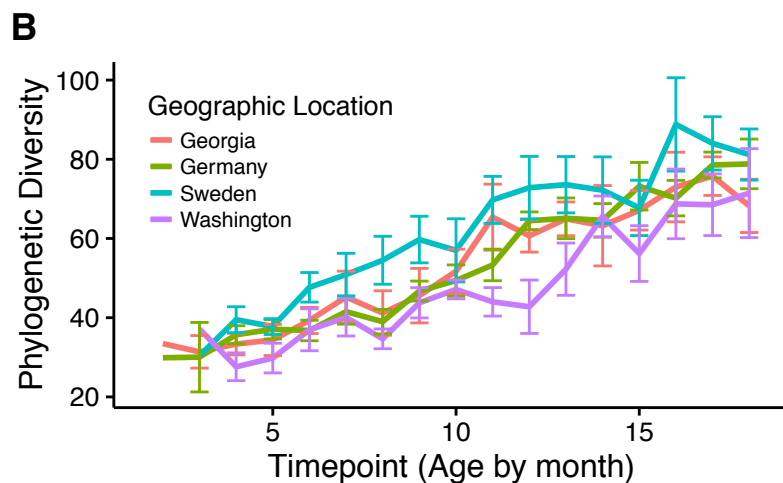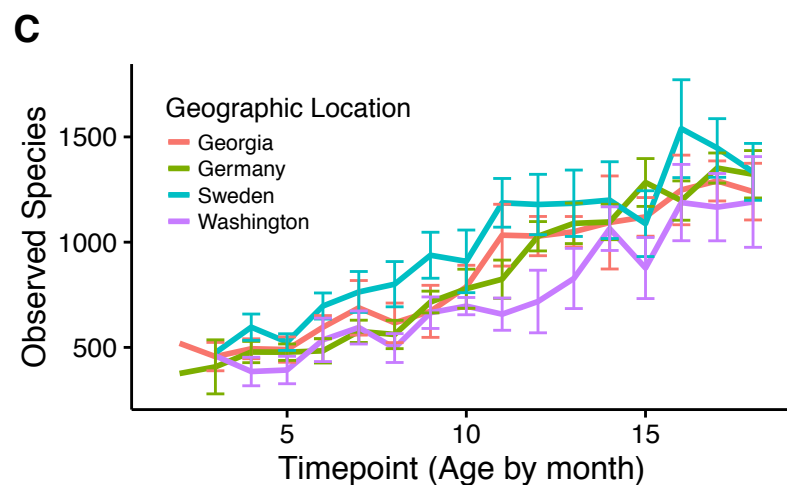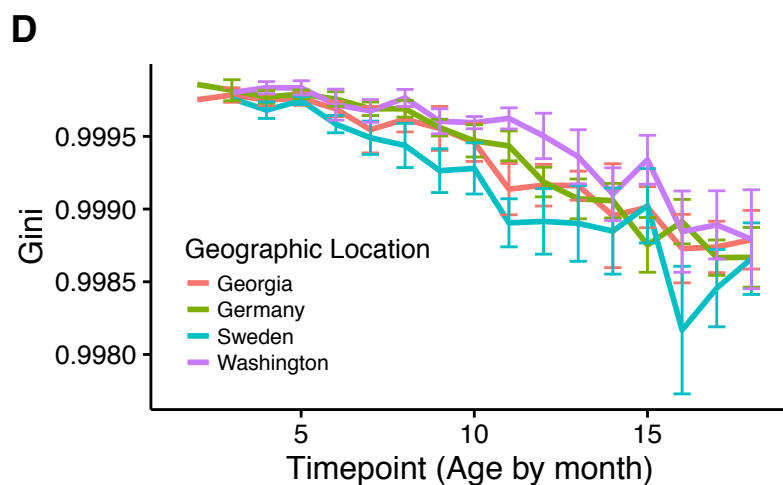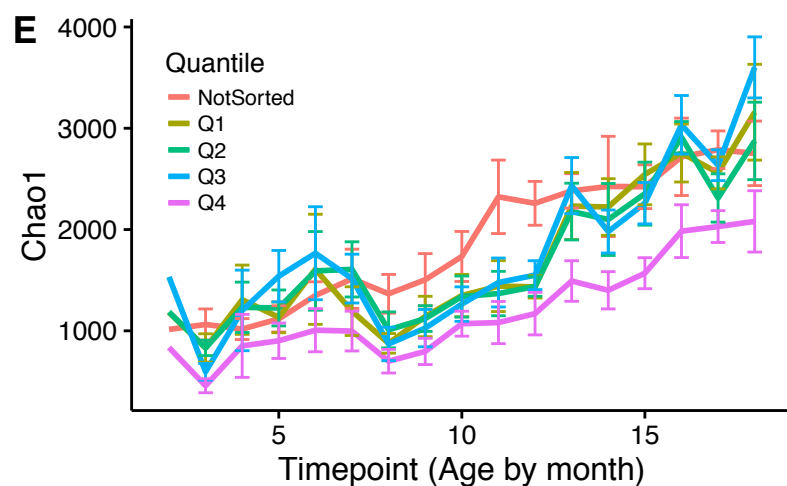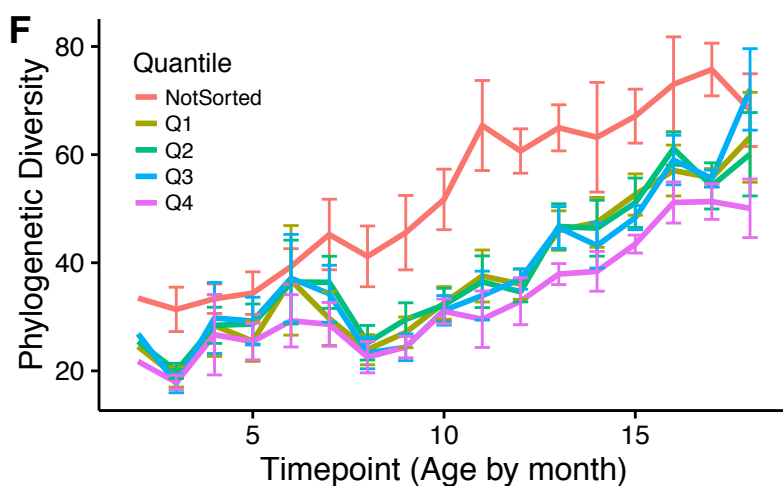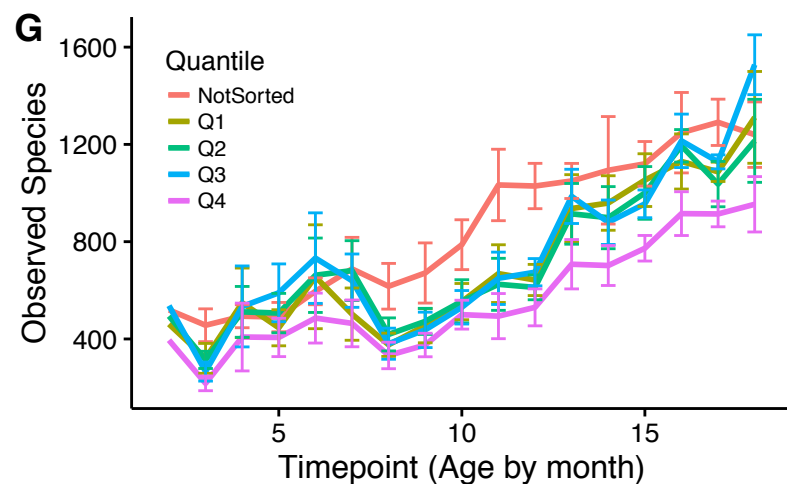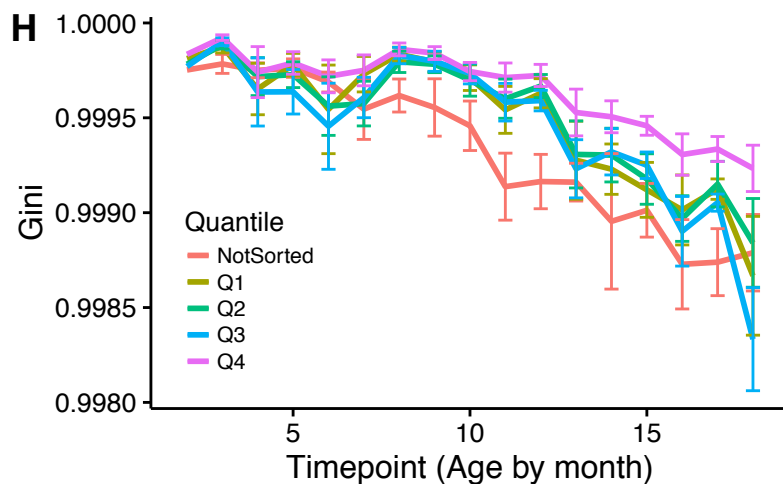

Supplement: FIG S2 [file mSystems.00612-19-sf002.pdf]

Sample ID

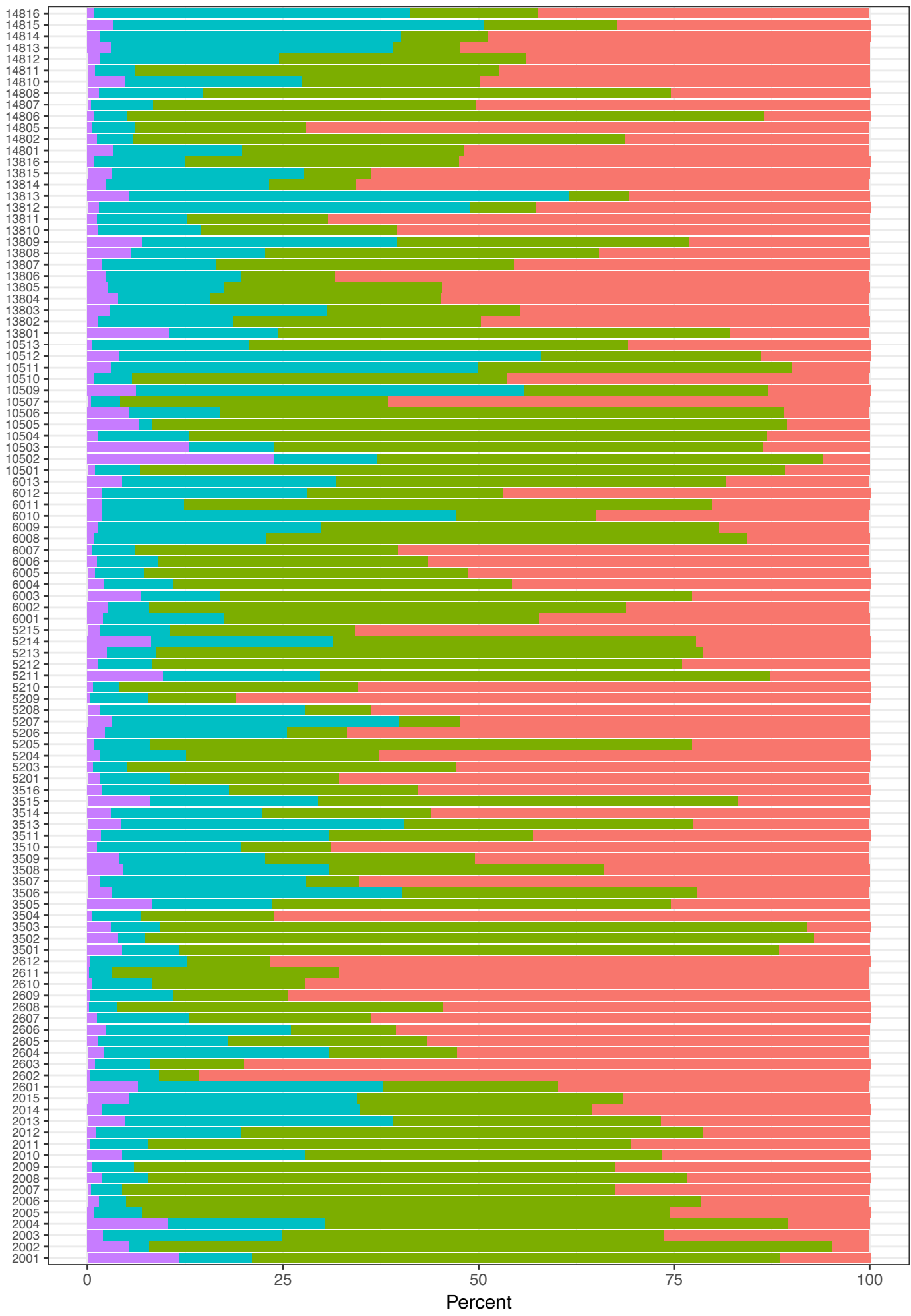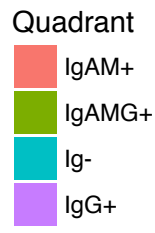

Supplement: FIG S3 [file mSystems.00612-19-sf003.pdf]

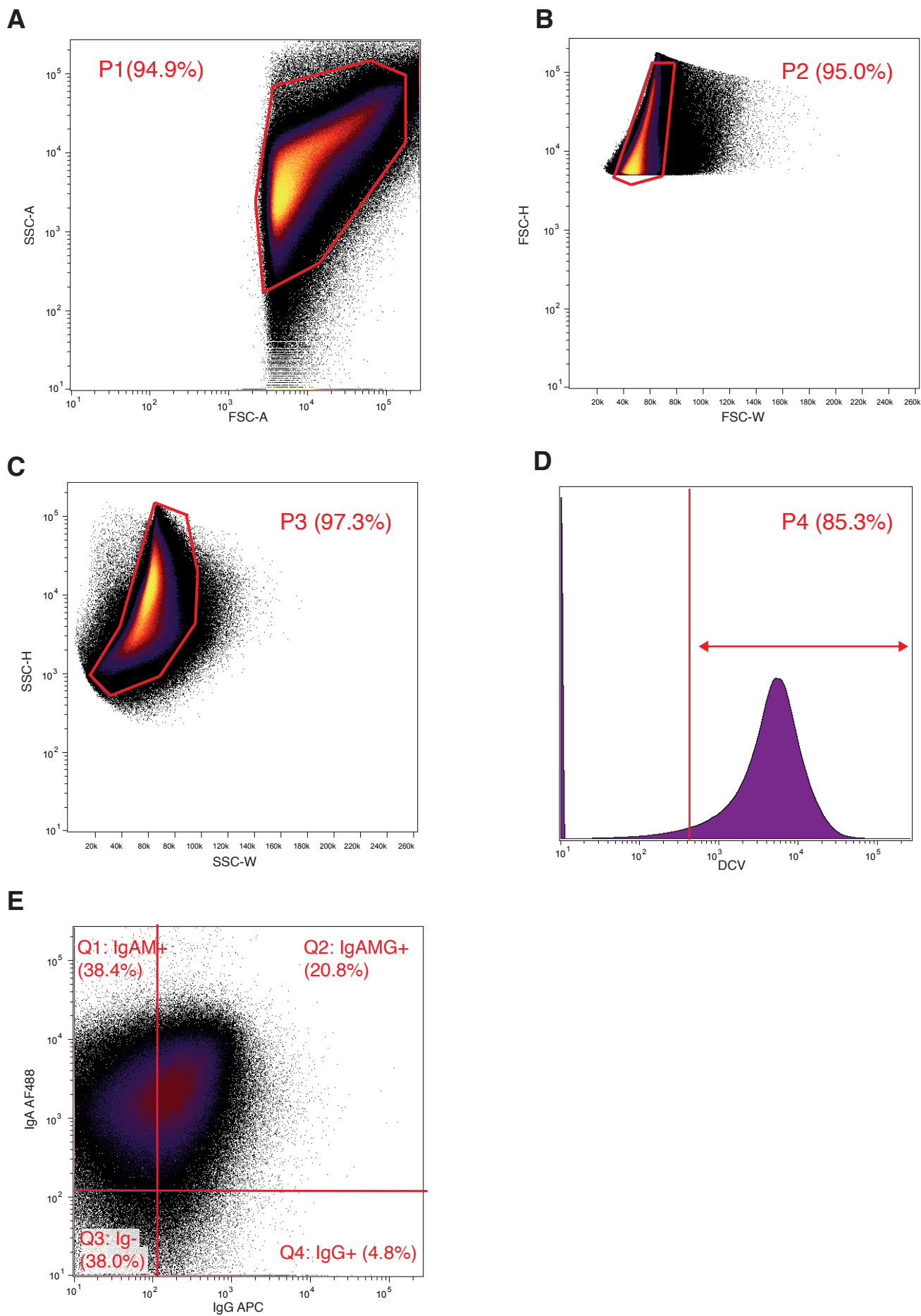

Supplement: FIG S4 [file mSystems.00612-19-sf004.pdf]

**A**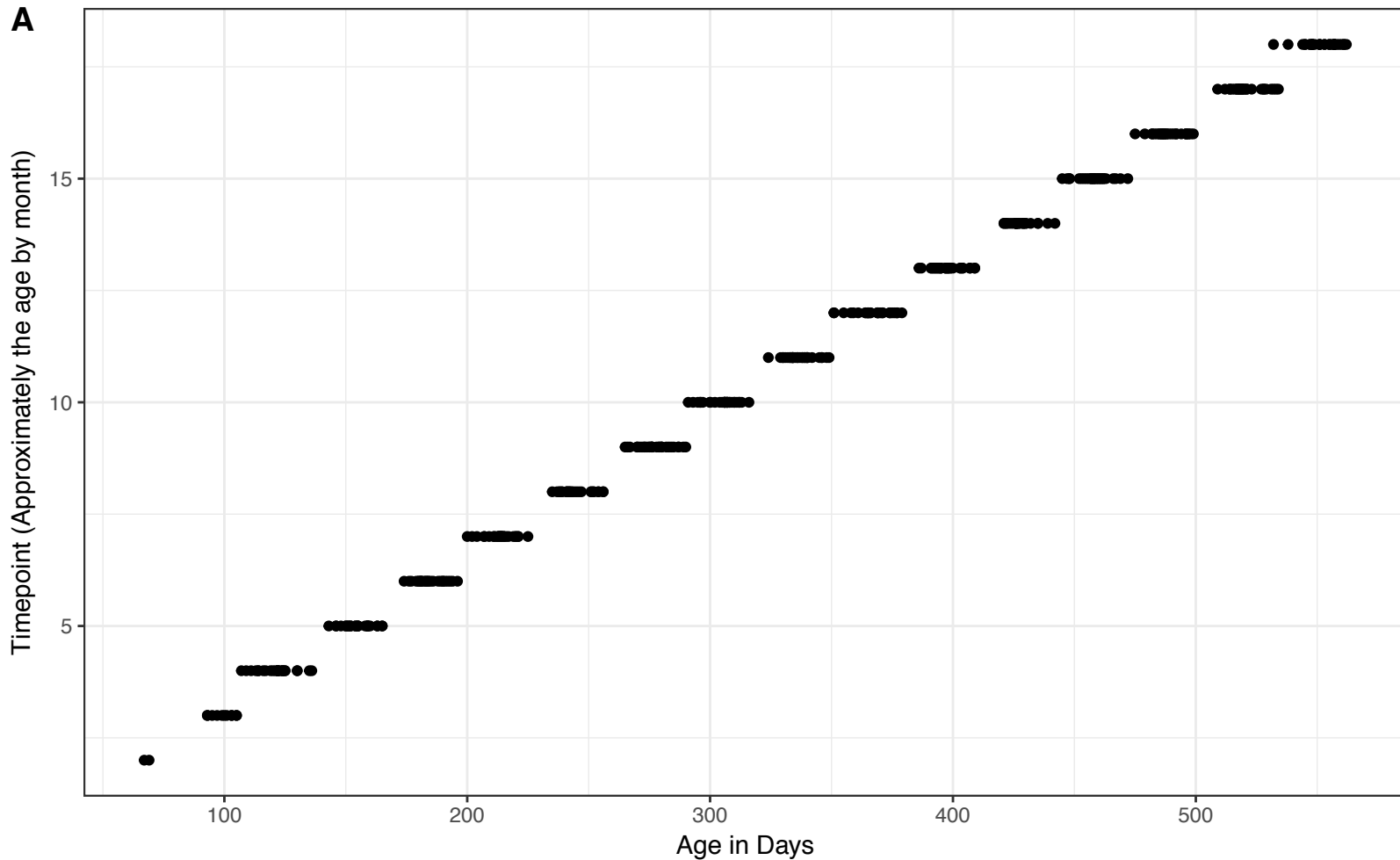**B**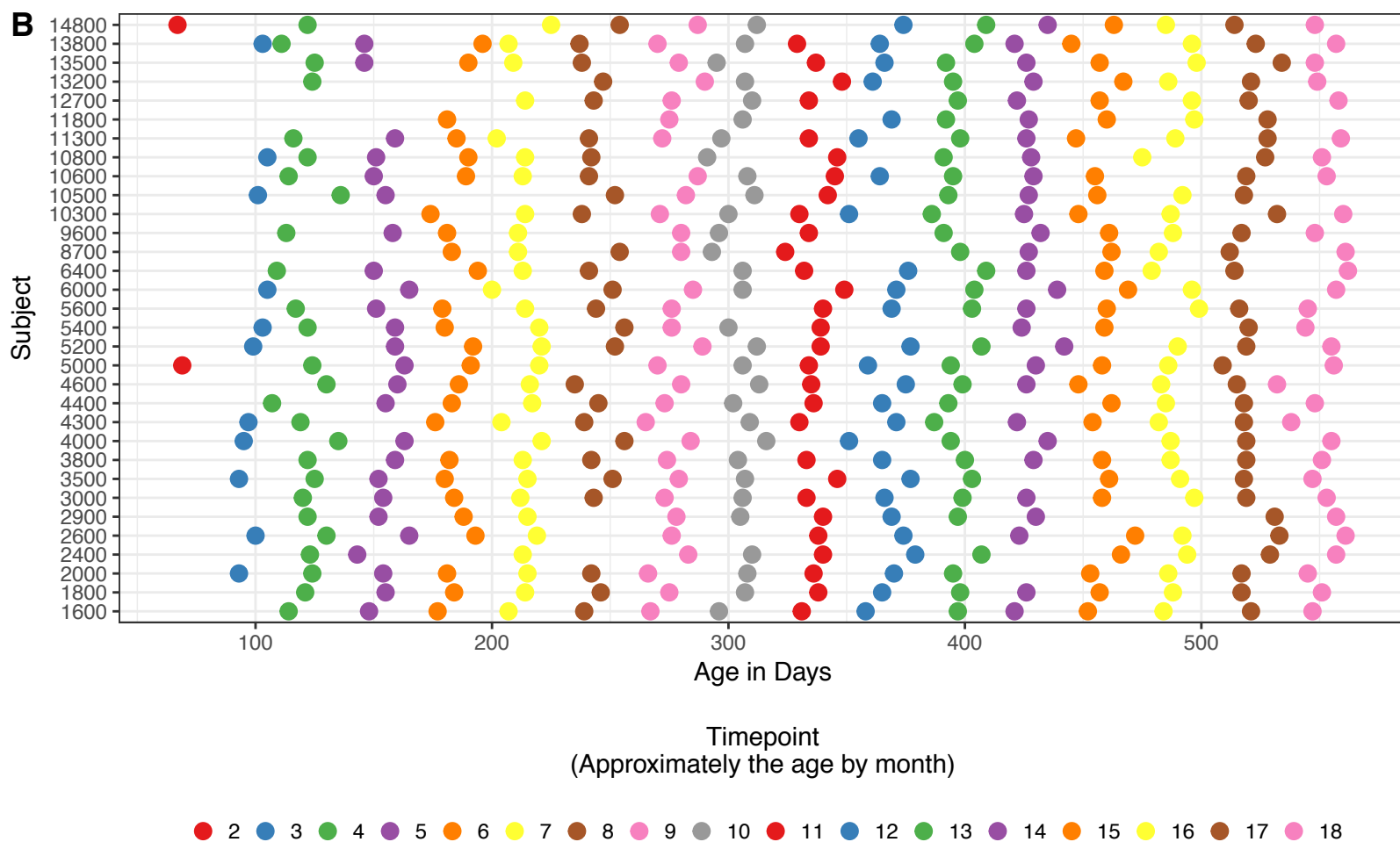

Supplement: FIG S5 [file mSystems.00612-19-sf005.pdf]
